# Polydopamine Nanomaterials for Overcoming Current Challenges in Cancer Treatment

**DOI:** 10.3390/nano13101656

**Published:** 2023-05-17

**Authors:** Shahinur Acter, Michele Moreau, Robert Ivkov, Akila Viswanathan, Wilfred Ngwa

**Affiliations:** Department of Radiation Oncology and Molecular Radiation Sciences, Johns Hopkins School of Medicine, Baltimore, MD 21287, USA

**Keywords:** polydopamine, multifunctional nanoparticle, cancer treatment, nanocarrier, photothermal therapy, radiation therapy, immunotherapy, synergistic treatment

## Abstract

In efforts to overcome current challenges in cancer treatment, multifunctional nanoparticles are attracting growing interest, including nanoparticles made with polydopamine (PDA). PDA is a nature-inspired polymer with a dark brown color. It has excellent biocompatibility and is biodegradable, offering a range of extraordinary inherent advantages. These include excellent drug loading capability, photothermal conversion efficiency, and adhesive properties. Though the mechanism of dopamine polymerization remains unclear, PDA has demonstrated exceptional flexibility in engineering desired morphology and size, easy and straightforward functionalization, etc. Moreover, it offers enormous potential for designing multifunctional nanomaterials for innovative approaches in cancer treatment. The aim of this work is to review studies on PDA, where the potential to develop multifunctional nanomaterials with applications in photothermal therapy has been demonstrated. Future prospects of PDA for developing applications in enhancing radiotherapy and/or immunotherapy, including for image-guided drug delivery to boost therapeutic efficacy and minimal side effects, are presented.

## 1. Introduction

Worldwide, cancer remains a leading cause of death and morbidity with a complex pathophysiology [1]. Standard of care includes surgery, radiotherapy, chemotherapy, and immunotherapy [2]. Though advances in cancer treatments have improved overall survival for patients diagnosed with some cancers, aggregate cancer death rates remain high [1]. The most significant barriers to improving cancer survival are inherent tumor heterogeneity and plasticity that enable drug evasion and drug resistance, respectively [3]. Though not a complete list, these factors are often couple with dose-limiting toxicities of anticancer agents that inhibit the complete eradication of disease [4]. Targeting drugs to the tumor is often considered to be highly relevant; yet, this goal remains elusive [4,5].

In order to overcome the limitations of cancer treatment, nanoparticle-based therapy approaches were introduced over two decades ago [6,7,8,9]. Since then, this field has grown rapidly [10,11,12,13]. A notable achievement is when paclitaxel albumin-bound nanoparticles received FDA approval for chemotherapy to treat various cancers, including lung, breast, and pancreatic, in 2005 [14,15]. Eventually, researchers extended work in cancer nanomedicine, devoting time to designing advanced nanoparticles, which could meet the challenges of cancer treatment by not only delivering chemotherapy drugs but also providing a range of important tools, such as in developing photothermal therapy, nanoparticle-aided radiation therapy, image-guided therapeutic drug delivery, photodynamic therapy, and immunotherapy [16,17,18]. However, there are limitations of nanoparticle-based systems for cancer therapy, such as slow cellular uptake, inconsistent intracellular distribution of drugs, interactions with or uptake by host immune cells, carrying sufficient quantities of drugs or molecules to targeted sites, circumventing multidrug resistance, and the emergence of long-term side effects, which have been demonstrated in vitro and in vivo studies [7,19,20,21].

Early reports of the tumor accumulation of protein colloids led to the development of the enhanced permeability and retention (EPR) paradigm that describes tumor uptake and the retention of nanoparticles [22,23]. Subsequently, discord between nanoparticle performance in clinical trials and post-approval clinical use with the expectations raised by preclinical models raised skepticism about the universal validity of the paradigms guiding the development of cancer nanomedicines [24,25,26]. More recent results indicate that the accumulation of nanoparticles in the tumor vascularized area, as well as penetration into and retention within the tumor microenvironment, depends on complex and incompletely understood interactions between the nanoparticle(s) and biology of the host. Specifically, nanoparticle accumulation and retention in tumors depend on the physical–chemical properties of the nanoparticles; the location, biology, and type of the tumor; the status of blood perfusion in tumors and surrounding tissues; and the immuno-biology of the host [27,28,29,30]. Nanoparticle interactions with immune cells dominate tumor retention and induce T-cell-mediated tumor suppression in breast cancer [30]. Therefore, the need to understand and develop biocompatible and biodegradable nanoparticles with desired physical–chemical properties to carry sufficient amounts of drugs with multifunctional properties for combined treatment in animal models that more faithfully recapitulate clinical scenarios is attracting attention in cancer nanomedicine [31,32,33,34].

Recently, in vitro studies on nanoparticle-based drug delivery systems demonstrate that the size and shape of particles have a profound impact on regulating their properties as drug carriers, particularly in cellular internalization and intracellular distribution [35,36,37]. According to the published work in the last few years, nanoparticles with anisotropic morphologies have become a fast-moving research topic in biomedicine [38]. In order to overcome the limitations associated with the regular isotropic particles within biomedical applications, a number of anisotropic morphologies have been engineered: triangles, dimple-shaped, cubes, rods, discs, stars, walnuts, bowls/cups, etc., [39,40,41,42,43,44]. Research in the field of nanoparticles-based drug delivery systems shows that due to the morphology of certain anisotropic particles, minimal repulsive interactions with cancer cells occur, thus accelerating their cellular uptake in cancer cells, thereby ensuring efficient and fast tumor penetration, which is an important property of an efficient drug nanocarrier [45,46,47,48]. In this field, polymer-based anisotropic particles have gained extra attention due to the flexibility in engineering their desired morphology and size, surface modification properties, biocompatibility, and colloidal stability [49,50]. Apart from the size and shape of the nanoparticles, the type of polymer material that nanoparticles are made of is a significantly important factor for not only their performance as nanocarriers but also their biocompatibility and biodegradability in the biological system as it is crucial in order to gain success in the field of cancer treatment [49,51]. Moreover, material with multifunctional properties, such as photothermal conversion efficiency, and with the property of being able to work as a radiosensitizer along with facile surface functionalization, is valuable in developing synergistic cancer therapies.

Among various materials, polydopamine (PDA) is attracting significant interest for its ability to form nanoparticles of various sizes and shapes, meaning they can be applied in a wide range of biomedical fields, including cancer treatment [52,53,54]. It is a nature-inspired insoluble polymer produced by the autoxidation of dopamine (monomer) in a basic environment [55,56]. Dopamine is one of the main neurotransmitters in the central nervous system, and it has been studied for its potential use in nanomedicine-based treatment of brain diseases [57,58]. PDA is a biocompatible and biodegradable polymer with a range of suitable properties for developing biomedical applications, including cancer nanomedicine [59,60]. Moreover, due to the dark color, PDA has the capability to absorb visible light and convert it to heat, and it is highly versatile for functionalizing material surfaces, meaning it offers the flexibility of loading drugs, molecules, etc [61,62,63,64]. Furthermore, functional groups such as catechol and amino groups in the structure of PDA give it unique reactivity and an efficient coating ability on nearly any surface [52]. Therefore, it offers the opportunity to modify surfaces with a range of non-metal and metal nanoparticles, including Si, Au, and Fe [65,66].

In a number of studies, PDA-coated Au nanostructures, including gold nanostars and gold nanorods, have been investigated for cancer treatment [67,68,69,70]. Herein, PDA works as both a reducing and capping agent to coat Au surfaces [71]. PDA-coated metal nanoparticles, including Au and Fe, have been successfully applied in studies for image-guided cancer diagnosis and therapeutic agents [72]. For example, Lin and colleagues demonstrate the efficiency of PDA-coated iron oxide (Fe_3_O_4_@PDA) nanocomposites for intracellular mRNA detection and multimodal imaging-guided photothermal therapy [73]. In this study, iron oxide serves as an image-contrast agent for MRI, which is composed of nanoparticles of iron oxide crystals coated in carbohydrates [73,74]. This has the capability to enhance MR images by altering the relaxation times of tissues in which the agent is present [73,74]. Perlman and co-workers studied the efficiency of Au/Cu@Polydopamine nanocomposites for CT-MRI contrast enhancement [75]. In their investigation, it has been observed that the PDA works as a reducing agent for both CuO NPs and HAuCl4 [75]. Studies show that Au-based nanoparticles work not only as vehicles for therapeutics delivery but also as imaging contrast agents, including for CT and nuclear imaging, fluorescence imaging, photoacoustic imaging, and X-ray fluorescence imaging [76]. Additionally, along with the flexibility of designing various sizes and shapes of PDA nanoparticles with outstanding drug loading capability, PDA is also available for targeted drug delivery [77]. By taking the advantages of abundant catechol and amino groups on PDA surfaces, they are capable of the modification of various targeting ligands on the surface of PDA nanoparticles [67,77]. With proper utilization, PDA has become a widely used material in the research of developing multifunctional nanoparticles with image-guided and controlled drug delivery for synergistic cancer therapy with higher efficiency and minimal side effects.

The aim of this review is to summarize the studies that have been conducted on PDA, and to address current challenges in cancer treatment. A comprehensive overview of the types and properties of PDA nanomaterials will be presented, including what makes this material suitable for medical research, particularly in cancer nanomedicine. Finally, we present potential future research directions to address the current limitations of nanoparticle-based cancer treatment, such as image-guided immunotherapy and radiation therapy.

## 2. Various Types of PDA Nanostructures and Their Formation Method

**Figure 1:**
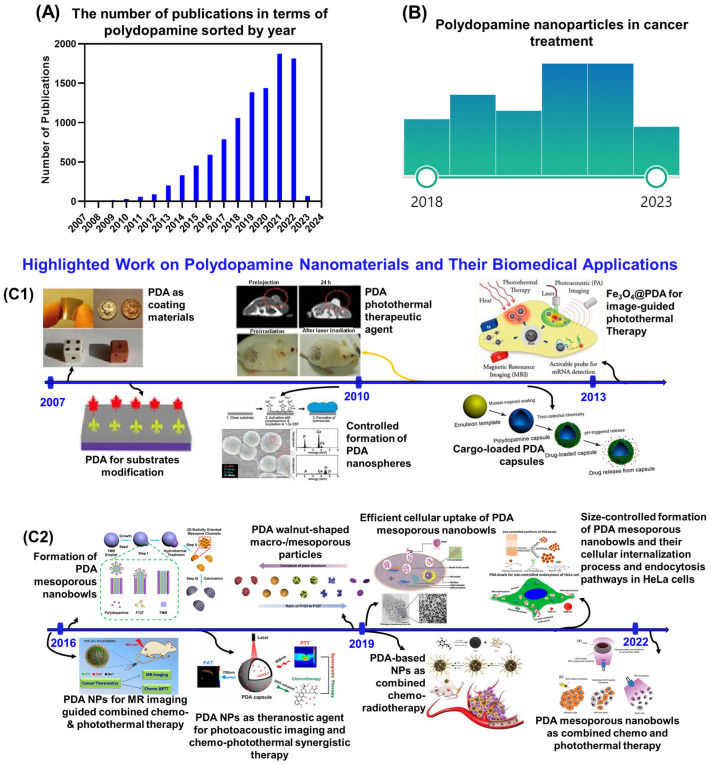
(**A**) The number of publications in terms of polydopamine sorted by year. Data were collected from the “Web of Science”. The word “polydopamine” is keyed into the “topic” search box (date of search: 5 January 2023). (**B**) Polydopamine nanoparticles applied in various cancer treatments in last five years (figure produced from PubMed search). (**C**) A brief timeline for the development of polydopamine in the form of coating materials and nanoparticles of various shapes and sizes, as well as their biomedical applications (**C1** (2007–2013) and **C2** (2016–2022)) [43,44,46,56,63,73,80,86,87,88].

PDA is a melanin-like naturally produced neurotransmitter, which is derived by the oxidant-induced polymerization of dopamine (monomer) in basic pH conditions [78]. However, the polymerization mechanism and the structure of PDA have not been fully understood. Currently, it is assumed that an indole skeleton is formed by oxidative ring closure in the first step of this polymerization process, followed by connecting the monomer units through dehydrogenative C-C bond formation [79]. The structure of PDA offers flexibility in the formation of various sizes and shapes of particles and functionalization due to the presence of carbonyl moieties, which act as electrophilic sites for amino- or mercapto-nucleophiles [62,80,81]. These functional groups, such as catechol, amine, and imine, make PDA valuable due to their extraordinary properties of working as the starting points for covalent modification with the desired molecules and serve as the anchors for loading drugs or hydrophilic polymer grafting (i.e., PEGylation) or surface modification with metal ions [63,82,83,84]. Therefore, PDA has been identified as an ideal coating material, and has rapidly been incorporated into a wide range of applications in various fields including medical research, particularly in cancer treatment [70,85]. From the timeline of PDA research (Figure 1), in 2007, PDA was used as a coating material for diverse surfaces for the first time. Ever since, it has attracted enormous attention as a versatile coating [61]. In 2010, cargo-loaded PDA capsules were introduced [61]. After that, a dramatic development was observed in the designing of PDA-derived nanoparticles when investigating their wide application in various biomedical fields, shedding light on sustainable nanomaterial-based drug release systems that have been tentatively applied, particularly in cancer treatment.

### 2.1. PDA Core–Shell Structure

**Figure 2:**
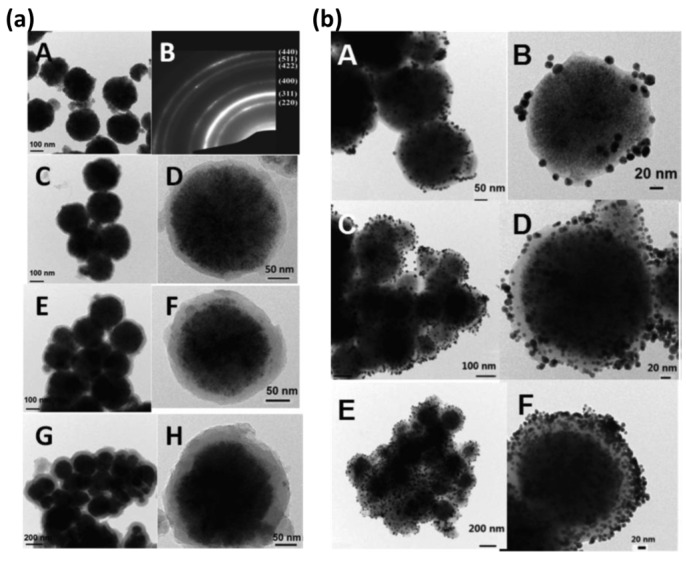
Transmission electron microscope (TEM) images of the nanoparticles. (**a**) (**A**,**B**) Fe_3_O_4_ nanoparticles A and SAED of Fe_3_O_4_ nanoparticles, (**C**,**D**) with PDA coating for one coating layer, two coating layers (**E**,**F**), and three coating layers (**G**,**H**), herein, (**D**,**F**,**H**) are high magnified view of (**C**,**D**,**E**) respectively. (**b**) Deposition of Au nanoparticles on PDA surface of Fe_3_O_4_@PDA nanoparticles (**A**–**F**), which increased with PDA shell thickness, herein, (**B**,**D**,**F**) are high magnified view of (**A**,**C**,**D**) respectively. Reprinted with permission from [84].

PDA has the ability to adhere to surfaces and also serve as a reducing and stabilizing agent due to its surface-active functional groups. For instance, Zhao and co-workers have developed a well-defined core–shell nanostructure of Fe3O4@PDA nanoparticles by utilizing the facility of having catechol groups in the structure of dopamine through a dehydration process [83]. Herein, Fe_3_O_4_ nanoparticles were successfully coated with a thin PDA-shell layer of 15 nm after a single coating (shown in Figure 2a(C,D)) due to the bonding created between hydroxyl groups on the Fe3O4 surface and catechol groups present on the PDA surface [83]. It is also observed that the thickness of the PDA layer can be increased with repeated coating following the same procedure (Figure 2a(C,H)) [83]. Due to the surface property of PDA, Fe3O4@PDA core–shell nanoparticles serve as catalyst support for the deposition of Au nanoparticles on the surface [83]. Herein, the PDA surface works as both a reducing and capping agent [83]. The morphology of the Fe3O4@PDA@Au nanoparticles has been characterized by using TEM imaging analysis (Figure 2b) [83]. As demonstrated, the core–shell thickness of PDA has a vital role in the deposition of Au nanoparticles on the surface of Fe3O4@PDA nanoparticles, as with the increased thickness of the PDA shell, Au nanoparticles deposition increased significantly (Figure 2b(A–F)) [83].

This multifunctional nanoparticle can be used in various biomedical applications. Previous studies suggest that magnetic nanoparticles with an iron oxide core could detect hemozoin by using surface-enhanced-resonance Raman spectroscopy (SERRS) [89,90]. Hemozoin is a by-product of malaria infection in erythrocytes, which has been identified as a biomarker for the diagnosis of malaria at an early stage [90]. Moreover, Lin and colleagues reported that these multifunctional Fe_3_O_4_@PDA core–shell nanocomposites have the ability to act as theragnostic agents for the detection of intracellular mRNA, can be used in multimodal image-guided photothermal therapy by utilizing the properties of PDA (near-infrared absorption, high fluorescence-quenching efficiency, surface functionalization with biomolecules, etc.), highlighting the magnetic resonance imaging property of the Fe_3_O_4_ core [73].

Evidence shows that PDA coating improves the intracellular uptake efficiency, biodistribution, and biocompatibility of the nanoparticles. Therefore, PDA has been used as a coating material for various surfaces, including Au, to enhance their capability as a drug nanocarrier with multifunctionality and minimal toxicity in the biological system. For example, Liu et al. successfully modified Au nanoparticles with well-controlled PDA monolayers to synthesize Au@PDA-shell nanoparticles [91]. They observed the different cellular internalization behavior of PDA-coated Au nanoparticles in vivo, which showed stability within the intracellular environment of the liver and spleen for six weeks without any notable histological toxicity to the main organs of mice for a long period of time [91]. An outstanding stability of PDA-coated nanostructures in vivo has been reported, and they were was found to be stable within the immune cells [91]. This is important information for designing PDA-based nanoparticles for use in cancer immunotherapy.

### 2.2. PDA Hollow Structure

**Figure 3:**
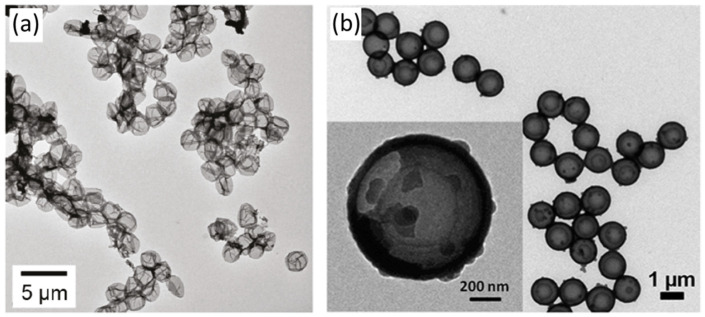
(**a**) Differential interference contrast microscopy (DIC) images for (**a**) PGAPDA15 and (**b**) PGAPDA25 capsules. Reprinted with permission from [60,88], respectively.

PDA-based or PDA-coated hollow capsules have gained attention as drug nanocarriers for enhancing the therapeutic effects of cancer treatment [92]. Caruso and co-workers synthesized PDA capsules for the first time in 2011, tailoring biodegradable capsules derived by the polymerization of dopamine [60]. They reported a synthesis method where PGA (poly L-glutamic acid) polymer is conjugated with DA through an amination process with various degrees of functionalization, followed by assembling the PGAPDA onto the silica particles and a core removal step resulting in biodegradable PGSPDA capsules (Figure 3a) [60]. For the fabrication of PDA hollow capsules, coating PDA on various templates followed by core removal is a versatile procedure, where template (either soft or hard) plays a vital role in well-defined shape formation [60]. However, a soft template is considered a better option in some situations to avoid harsh chemical reagents, including acids and organic reagents [88]. For instance, Caruso and co-workers reported a formation method of PDA capsules using soft-template dimethyldiethoxysilane (DMDES) emulsion droplets for the formation of PDA capsules, where the shell thickness and the size can be tuned (Figure 3b) [88]. They reported loading anticancer drugs into the PDA capsule during the formation process in the emulsion template system [88]. In this study, the PDA coating was performed via an in situ polymerization method before the formation of the capsule. The loaded drugs were found intact inside the capsules after the removing of the template using aqueous ethanol. Later on, pH-dependent fluorescence release via PDA capsules was reported by the same group, where they observed higher fluorescence intensity at pH 3 in the system, suggesting this capsules can be applied for pH-triggered drug release applications [88]. Inspired by this study, recently, Acter et al. demonstrated higher doxorubicin (DOX) release from PDA mesoporous nanobowls in an acidic environment (at pH 5.5) [63]. It has been suggested that an acidic environment is favorable for the disruption of π–π stacking between PDA mesoporous nanobowls and DOX due to the protonation of the amine group of PDA [63,93].

### 2.3. PDA Spheres

**Figure 4:**
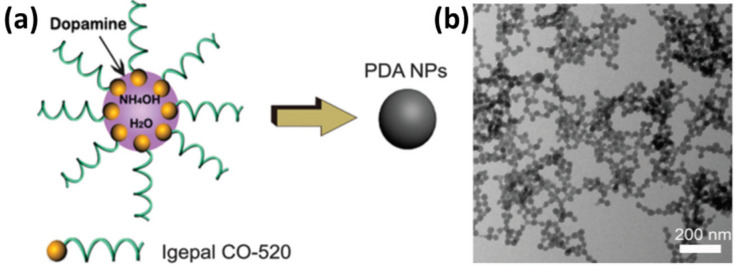
(**a**) Schematic illustration of the synthesis process of the PEG–Fe–PDA NPs; the illustration is not drawn to scale. (**b**) TEM micrograph of the PDA NPs (25 2.0 nm in diameter, obtained with 7.5 mL of a dopamine hydrochloride aqueous solution). Reprinted with permission from [94].

PDA spheres or spherical-shape nanoparticles with tunable size can be synthesized without any template. Challenges when using a template include the use of harsh chemical reagents for removing the hardcore template, which is a real concern for the soft-template method. One of the limitations is reproducing particles of similar sizes, shapes, and surface properties. A facial synthesis method of PDA nanoparticles has been reported by Liu et al., where PDA nanoparticles in various sizes were synthesized in ethanol–water mixtures in the presence of ammonia (Figure 4) [94]. The particle sizes can be controlled by tuning the reaction mixture, such as increasing the temperature of the reaction medium, leading to smaller size particles [94]. Additionally, the concentration of ammonia and dopamine has been found to have a noticeable impact on controlling the size of the nanospheres [94].

### 2.4. PDA Anisotropic-Shaped Nanoparticles

**Figure 5:**
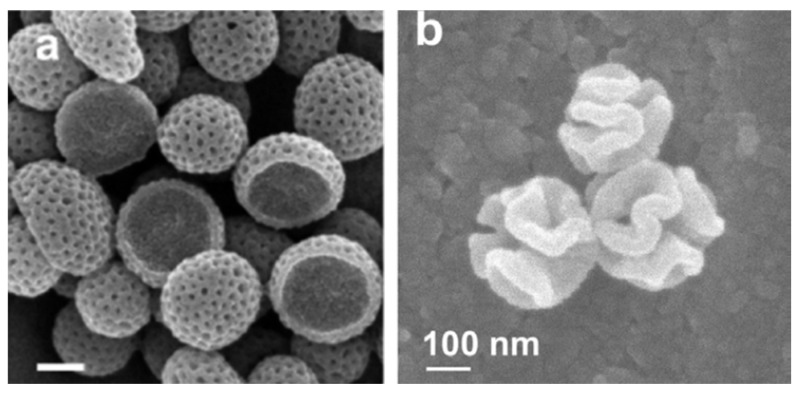
FESEM images, (**a**) PDA mesoporous nanobowls and (**b**) PDA walnut-shaped nanoparticles. Reprinted with permission from [44,43], respectively.

In the development of particles-based drug delivery systems, it is well established that the properties of the carriers, such as size, shape, and surface characteristics, are important factors in altering cellular internalization, cell viability, hemocompatibility, and biodistribution in the biological system [46,95,96]. A number of studies have been performed on designing various shapes and sizes of PDA nanoparticles. For example, Guan and co-worker developed an emulsion-induced interface anisotropic assembly method to synthesize bowl-like PDA mesoporous nanoparticles in 2016 [44]. The diameter of the nanostructures is ~210 nm, with ~8 nm mesopores and a ~70 nm cavity (Figure 5a) [44]. There are well-controlled radially oriented mesochannels, and center-to-center distance between the two adjacent mesochannels is ∼21 nm [44]. A couple of years later, the same group synthesized PDA walnut-shaped particles with controllable macro-/mesoporous properties by using a pore architecture manipulation process (Figure 5b) [43]. In 2020, shape-dependent cellular internalization studies of PDA nanoparticles were performed by Acter et al. for the first time, where they observed a dramatically faster cellular internalization of bowl-shaped PDA mesoporous nanoparticles in HeLa cells (cervical cancer cells) compared with their spherical counterparts [46].

**Figure 6:**
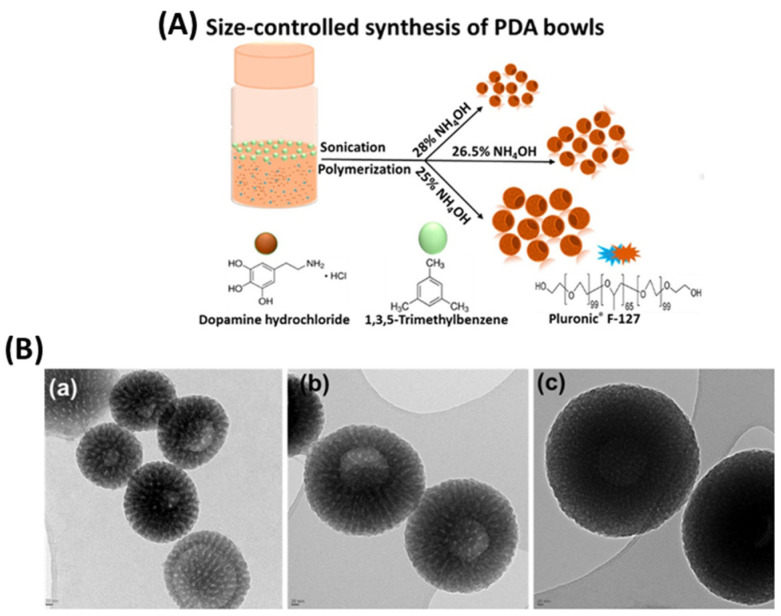
(**A**) Schematic diagram shows the impact of ammonia on the size of the PDA bowl-shaped mesoporous nanoparticles. (**B**) TEM images of PDA bowls at around (**a**) 180 nm, (**b**) 350 nm, and (**c**) 520 nm. Reprinted with permission from [80].

Inspired by the advantages of PDA bowl-shaped mesoporous nanoparticles over their spherical counterparts, in their follow-up study, they conducted the size-controlled formation of PDA bowl-shaped mesoporous nanoparticles with tunable cavity and mesopore diameters [46,80]. In a range of experiments, the impact of each reaction component of the formation method was closely investigated, and the sole contribution of each reaction parameter in the development of the PDA mesoporous nanobowls established precise experimental conditions for the size-controlled formation of PDA mesoporous nanobowls with well-defined and reproducible physicochemical properties (Figure 6) [80]. In 2016, Chen and co-workers introduced mesoporous PDA nanoparticles, synthesized by a facial approach, which involves the assembly of primary PDA nanoparticles and surfactant (Pluronic F127)-stabilized emulsion droplets on water/1, 3, 5-trimethylbenzene (TMB) interfaces [97]. In this formation method, the key part is the full utilization of the π–π stacking interactions of PDA structures and the π-electron-rich trimethylbenzene [97]. One of the biggest advantages of the mesoporous morphology is the higher absorption of model dye molecules, suggesting the potential to carry larger amounts of drugs or molecules compared with their spherical counterparts [97].

## 3. Potential of Polydopamine Nanoparticles for Cancer Treatment

A nature-inspired biopolymer, PDA has gained enormous attention in the development of innovative modes of cancer treatment with minimal side effects due to its range of excellent properties, including biocompatibility and biodegradability [67]. In a number of studies (shown in Figure 1), this material has been chosen in designing multifunctional nanoparticles considering its extraordinary active surface functionalization property, which allows it to bind with a range of molecules and materials; strong NIR absorption characteristics; and flexibility in the formation of nanoparticles in various sizes and shape [55,62,80]. A larger body of published work on PDA demonstrated that it has been used in studies for various applications, including drug delivery systems and diagnosis, as well as combined chemo- and photothermal therapy to treat cancer [63,67,70,81]. In the second part of this review, the efficiency and potential of PDA nanomaterials in order to meet the current challenges of nanoparticles-based cancer treatment have been summarized with the future prospects of this material for immunotherapy and radiation therapy in cancer treatment.

### 3.1. PDA for Drug Delivery and Photothermal and Photodynamic Therapy

A range of studies have been published on PDA in the form of nanoparticles, nanocapsules, and coating material for drug delivery and photothermal therapy for cancer treatment [62,70]. Taking the advantages of the adhesive property of PDA, it offers the potential to load both hydrophilic and hydrophobic types of drugs and provides sustained and pH-responsive release in the intracellular environment [84,98]. Pada et al. evaluated the molecular glue function of PDA by using it to carry sufficient amounts of hydrophilic and hydrophobic anticancer drugs [98]. In another study, Zheng et al. reported PDA-coated nanocarriers for their efficiency in loading and releasing hydrophilic drugs [84]. They demonstrated that due to π–π stacking interactions between the abundant aromatic rings of PDA and the aromatic backbones of drugs, it has high loading capacities, such as 380 μg/mg for doxorubicin hydrochloride (DOX) and 320 μg/mg for calcein [84].

**Figure 7:**
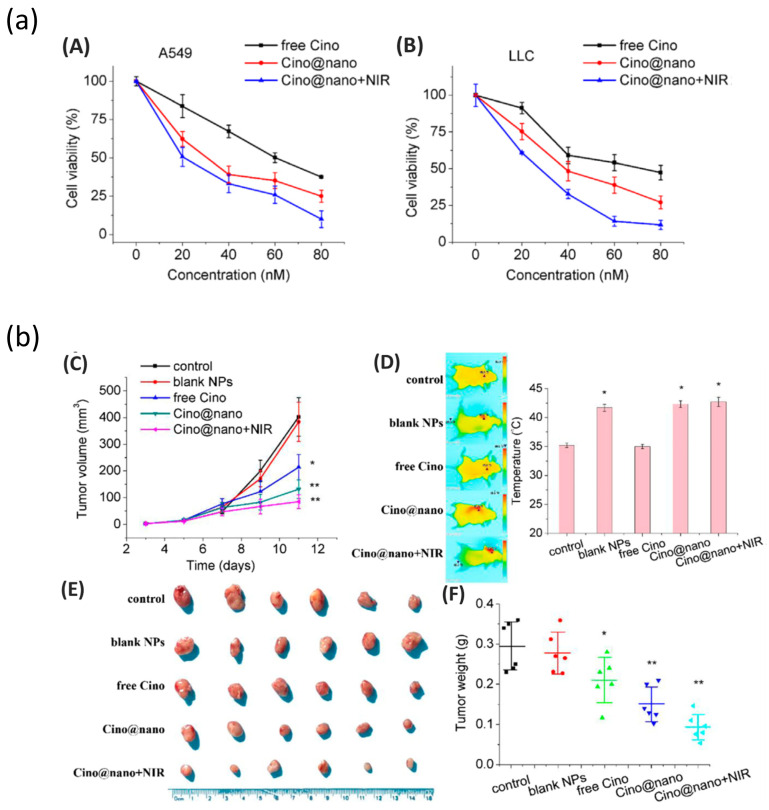
(**a**) In vitro antitumor efficacy of Cino nanomedicine in lung cancer cells. (**A**) Viability of A549 cells after incubation with various concentrations of free Cino, Cino-loaded PDA nanomedicine, and Cino-loaded PDA nanomedicine with NIR treatment. Data are presented as mean ± SD (standard deviation, n 4). (**B**) Viability of LLC cells after incubation with various concentrations of free Cino, Cino-loaded PDA nanomedicine, and PDA nanomedicine with NIR treatment (2 W cm^−2^, 5 min). Data are presented as the mean ± SD (standard deviation, n 4). (**b**) In vivo antitumor efficacy of Cino-loaded PDA nanomedicine. (**C**) Tumor volume growth curves, (**D**) optothermal response, (**E**) tumor photo, and (**F**) tumor weight of LLC tumor-bearing mice after systemic administration of saline, blank NPs, free Cino (1 mg/kg), Cino-loaded PDA nanomedicine (1 mg/kg of Cino), and Cino-loaded PDA nanomedicine (1 mg/kg of Cino) treated with 808 NIR laser (2 W cm^−2^, 5 min). Data are presented as the mean ± SD (standard deviation, n 6); * *p* < 0.05, ** *p* < 0.01. Reprinted with permission from [99].

**Figure 8:**
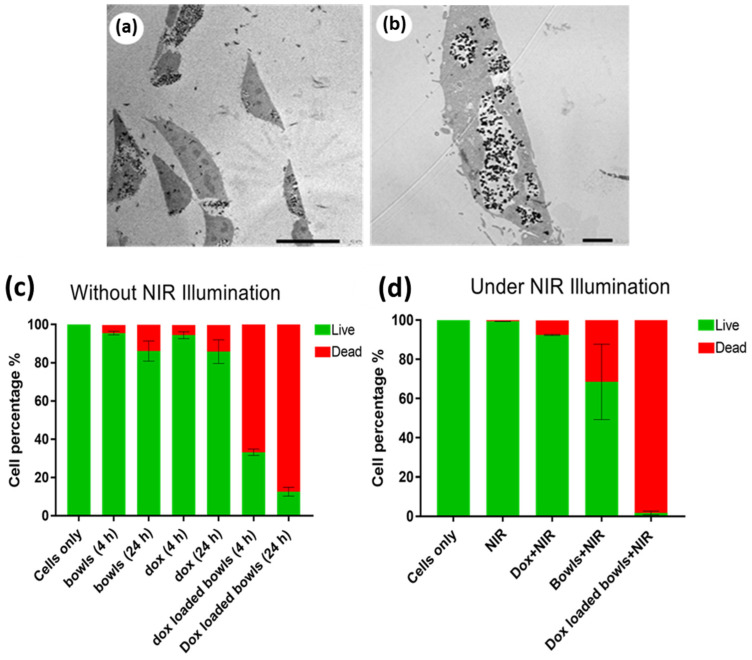
(**a**,**b**) TEM images of HeLa cells showing uptake of bowl-shaped polydopamine mesoporous nanoparticles; (**b**) is the high magnified view. (**c**,**d**) showing the impact of various treatments on HeLa cells without and with NIR illumination (1 W cm^−2^, 5 min), respectively. Green and red bars represent live and dead cells, respectively. Reprinted with permission from [46,63], respectively.

**Figure 9:**
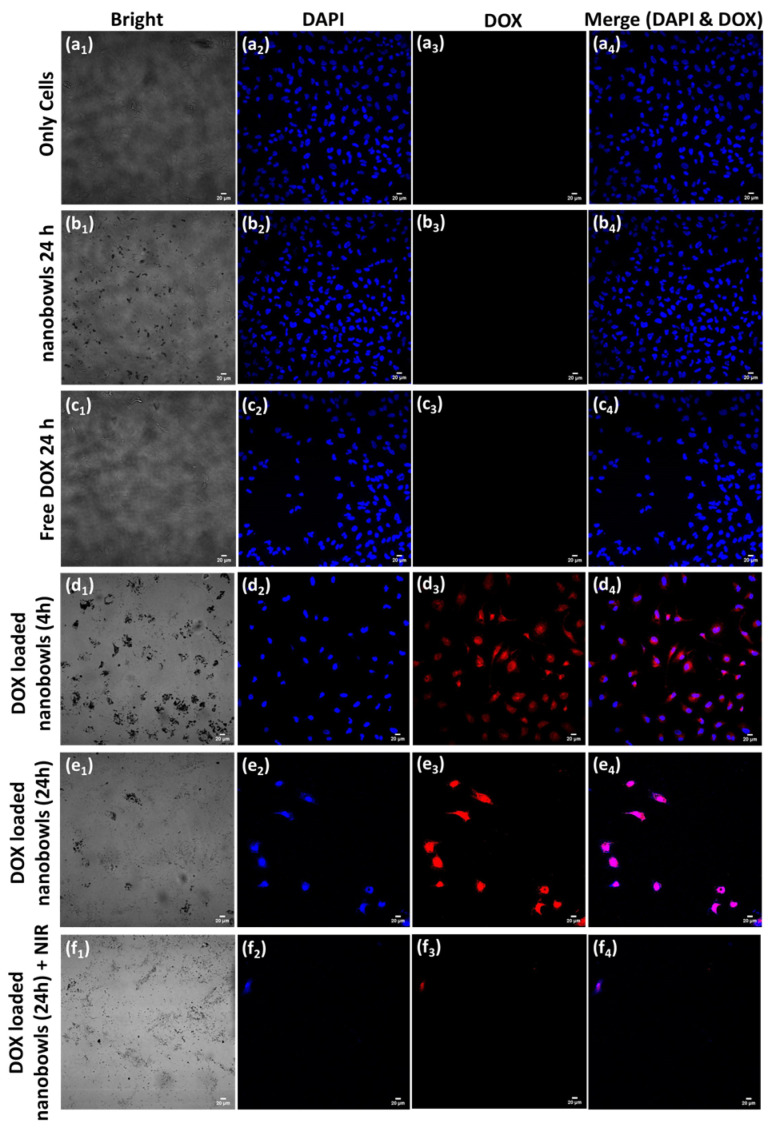
Confocal fluorescence images of HeLa cells after various treatments, herein, a_2_ to a_4_ only cells, b_1_ to b_4_ cells incubated for nanobowls for 24 h, c_1_ to c_4_ cells incubated with free DOX for 24 h, d_1_ to d_4_ cells incubated with DOX loaded nanobowls for 4 h, e_1_ to e_4_ cells incubated with nanobowls for 24 h and f_1_ to f_4_ cells were treated with NIR after 24 h of incubation with DOX loaded nanobowls. NIR illumination for 5 min, 1 W cm^−2^. Scale bars represent 20 µm. Reprinted with permission from [63].

A number of investigations that combined PDA nanoparticles with chemo- and photothermal therapy (PTT) for cancer treatment reported that PDA displays much greater photothermal conversion efficiency (40%) than that of previously reported PTT agents, such as Au nanorods (22%) [62]. Advantages such as the tunable size and shape of the particle, strong adhesive surface properties, and strong photothermal conversion efficiency makes PDA an effective material for the synergistic combination of chemo-photothermal therapy with NIR light irradiation to treat various types of cancer with high efficiency. For example, in 2017, Zhu and co-workers showed the efficiency of PDA spherical-shape nanoparticles (~200 nm) for combined photothermal and chemotherapy in killing HeLa (human cervical cancer) cells in vitro [62]. Li et al. recently reported success with PDA nanomedicine as part of a multimodal therapy to treat lung cancer with photothermal effects inhibiting tumor cell proliferation in vitro and in vivo [99]. They demonstrated the pH-responsive and NIR-irradiation-triggered drug release properties of PDA particles, which effectively work as anticancer agents and photothermal therapeutics in inhibiting tumor cell proliferation in both in vitro and in vivo studies, as shown in Figure 7 [99]. In other examples, Xing and colleagues demonstrated the high payload of anticancer drug in PDA mesoporous nanoparticles, showing its strong absorption of NIR light, which it converted into fatal heat to kill cancer cells [81]. This is considered a potential combined treatment system for synergistic chemo- and photothermal therapy to treat cancer with multidrug resistance [81]. Mesoporous surfaces have also been found to be beneficial in carrying drugs over their nonporous counterparts [81]. In 2022, considering the higher cellular uptake efficiency of PDA cup- or bowl-shaped mesoporous nanoparticles over their spherical counterparts, Acter et al. reported that bowl- and cup-shaped PDA mesoporous nanoparticles (PDA nanobowls) showed high efficiency as drug nanocarriers [46,63]. In this study, they also demonstrated the potential of this anisotropic nanostructure (PDA mesoporous nanobowls) in combining chemo- and photothermal therapy to treat cancer by killing cervical cancer cells (HeLa cells) in vitro [63]. To be more specific, due to the combined chemo- and photothermal treatment of HeLa cells using PDA nanobowls, nearly 100% cell death occurred in vitro [63]. As shown in Figure 8, PDA nanobowls were broadly distributed in the intracellular environment without any sign of aggregation (Figure 8a,b), which is crucial in order to gain success as drug carriers [46]. The experimental results suggested that PDA mesoporous nanobowls can act as an efficient drug nanocarrier, showing the bowl’s capability in efficiently carrying anticancer drug molecules into cells (Figure 8c), as a significant amount of cell death occurred after 24 h of incubating drug-loaded PDA nanobowls with HeLa cells compared with free drugs [63]. Moreover, a combined approach of chemo- and photothermal treatment caused a significant number of cell death events in comparison with free anticancer drugs and without photothermal treatment (Figure 8c,d) [63]. In addition, an excellent photothermal conversion efficiency of PDA nanobowls under NIR illumination has been shown to kill nearly 65% of cells alone without any anticancer drugs, which clearly identifies the efficiency of PDA nanobowls as photothermal agents (Figure 8c,d) [63]. After a series of in vitro investigations on HeLa cells, including biochemical assay testing and confocal imaging analysis, a significantly higher cytotoxic effect of the combined treatments of anticancer-drug-loaded PDA nanobowls followed by NIR illumination on HeLa cells was observed in comparison with free anticancer drugs, as shown Figure 9 [63]. The obtained results suggest that PDA mesoporous nanobowls have capabilities not only as efficient drug nanocarriers but also promising candidates for the development of synergistic chemo- and photothermal therapy to conquer multiple-drug resistance. Additionally, a number of reports demonstrate the efficiency of polydopamine-based nanoparticles for combined photothermal (PTT)/photodynamic (PDT) methods to achieve a synergistic therapeutic effect in cancer treatment [100,101,102]. For example, Mao and co-workers reported on a functionalized walnut-shaped polydopamine nanomotor (PDA-PEG-ICG-Fe^3+^) for photothermal (PTT) and photodynamic (PDT) synergistic therapy to kill tumor cells [101]. Herein, taking advantage of electrostatic/hydrophobic interactions, the photosensitizer indocyanine green (ICG) was loaded into the nanoparticles, followed by being chelated with ferric ion (Fe^3+^), offering synergistic therapy to kill tumor cells with the combined energy of PTT and PDT [101].

### 3.2. Efficiency of PDA as Image-Contrast Agent, and in Immunotherapy and Radiation Therapy

In order to overcome current challenges and minimize the side effects of cancer treatment, the use of nanoparticles for image-guided therapy along with immunotherapy and radiation therapy has also attracted enormous research attention [103,104,105,106]. In a number of published studies, PDA has been demonstrated as an image-contrast agent, as well as a carrier of immunotherapeutics due its excellent surface properties [107,108,109,110]. There are studies where PDA has been demonstrated as an image-guided therapeutic agent and an image-contrast agent capable of delivering immunotherapeutics and enhancing chemo-radiotherapy [62,108,110]. Wang et al. reported the results of investigations using gadolinium–PDA nanoparticles as magnetic resonance imaging (MRI) contrast agents [111]. Their in vivo investigation demonstrated that multifunctional gadolinium–PDA-based nanoparticles were efficiently constructed and applied as a theragnostic nanoagent for dual-modality T1-weighted MRI/dye-tracing regional lymph nodes (RLNs), as well as the guided radical photothermal therapy of metastatic RLNs [111]. Gadolinium-based contrast agents are widely used for MRI because of their paramagnetic properties with seven unpaired electrons, and they have also been found to improve radiotherapeutic efficacy [112]. Herein, the strong metal binding capabilities of the catechol-based functional group of PDA allow it to bond with gadolinium [111].

**Figure 10:**
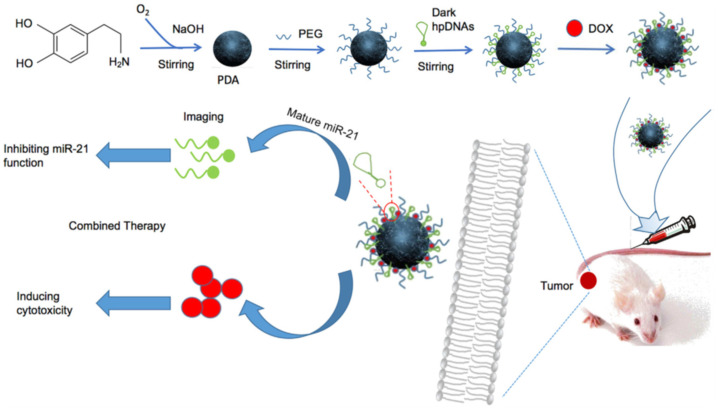
Schematic illustration of the development of PEG-PDA nanoparticles with co-loading Dox and FITC-labeled hpDNA for imaging of miRNA-21 and in vivo synergistic cancer treatment. Reprinted with permission from [113].

They have described gadolinium–PDA nanoparticles as contrast agents with several advantages, including in tumor imaging due to their enhanced permeability and retention effect [111]. Another group Mao et al. studied PDA-based theragnostic nanoprobes for the efficient detection of miRNA-21 and in vivo synergistic cancer therapy (Figure 10) [113]. In this study, fluorescein isothiocyanate (FITC)-labeled hairpin DNA (hpDNA) and an anticancer drug doxorubicin (DOX) were loaded into the PDA nanoparticles by π–π interactions and hydrogen bonding owing to the abundant catechol and amino groups on their surfaces [113]. FITC is a derivative of fluorescein used in wide-ranging applications in biological fields and is a broadly used material in image-guided therapy for cancer treatment, specifically recognizing miR-21 [113]. In the presence of miR-21, FITC-labeled hpDNAs bind to miR-21 followed by detachment from PDA-PEG to generate fluorescence [113]. Meanwhile, PDA, because of its excellent biocompatibility and biodegradability, as well as its extraordinary adhesion surface properties, is an ideal candidate for delivering immunotherapeutics in order to enhance immune response in tumor immunotherapy. It has been demonstrated that dopamine works as a neurotransmitter and also plays an important connector role between the nervous and immune system as an extracellular messenger, which is able to regulate the immune system by interacting with dopamine receptors on the immune cells [109]. Moreover, dopamine receptors are broadly distributed in the brain and peripheral tissues [109]. A previous investigation has shown antiangiogenic and anticancer activity via the activation of dopamine receptors on endothelial and tumor cells [109]. It has been suggested that dopamine is capable of activating resting effector T cells and suppress regulatory T cells [109].

**Figure 11:**
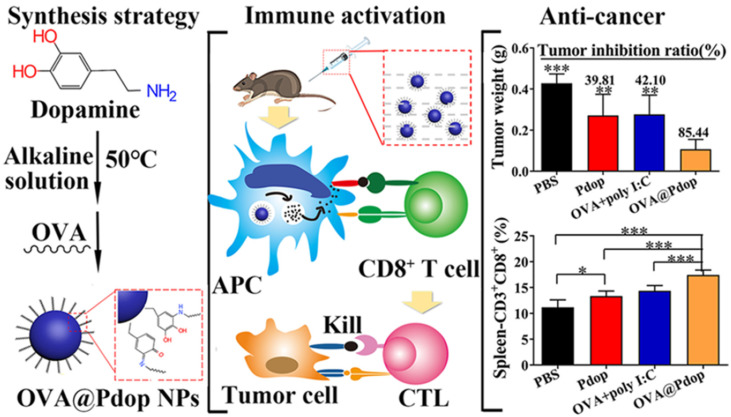
Shows the formation strategy of antigen-ovalbumin polydopamine nanoparticles (OVA@Pdop-NP) (**left**), immune activation of OVA@Pdop-NP (**middle**), and their antitumor effects (**right**). The differences between the groups were determined using one-way ANOVA followed by Tukey’s post-test. * *p* < 0.05, ** *p* < 0.01, *** *p* < 0.001. Reprinted with permission from [109].

All these properties make PDA an excellent material in antigen delivery applications to advance immunotherapy studies. PDA has been applied for synergistic approaches of photothermal immunotherapy in a number of studies, where it has been demonstrated as a multifunctional nano-platform for combined cancer therapy [114]. Furthermore, a range of studies have reported the potential of PDA-based nanoparticles in carrying only immumotherapeutics. For example, Wang et. al. investigated the efficacy of PDA nanoparticles as a subcutaneous antigen delivery vehicle in antitumor therapy in treating colon cancer (Figure 11) [109]. Herein, uniform PDA nanoparticles (Pdop-NPs) at around 200 nm were used to carry antigen-ovalbumin (OVA) by grafting onto the surface of the nanoparticles [109]. The loading capacity of OVA protein was 754 μg mg^−1^ [109]. As observed, OVA@Pdop-NPs exhibited higher cellular uptake and have shown flexibility in migrating to lymph nodes in vivo in comparison with the free OVA. In this study, in the colon-cancer-bearing model, they observed significantly suppressed tumor growth upon OVA@Pdop-NPs treatment by stimulating the CD8+ T-cell-mediated immune response and improving the immunosuppressive microenvironment within the tumor [109]. They showed the potential of Pdop-NP in developing cancer vaccines for the therapy of colon cancer and other types of cancers [109].

Due to the availability of highly reactive catechol and amine functional groups, PDA can allow further modification with a range of fluorescence dyes and metal coatings, such as Au, Cu, and Fe, which would extend its applications not only in image-guided therapy but also in combining other treatment modalities. PDA nanoparticles with metal coatings have been successfully applied as image-contrast agents for MRI and CT, which are significantly important tools for diagnosis and targeted cancer treatment. For example, Wang and colleagues synthesized iron oxide–PDA hybrid nanodots via the simultaneous fabrication of iron oxide nanoclusters by using nanoprecipitation followed by the polymerization of dopamine, resulting in hybrid nanodots for use in T1-weighter MR imaging and photothermal therapy [111]. These formatted PDA-based nanodots have shown potency in tumor targeting, a high photothermal conversion coefficient, and significant cellular uptake, resulting in complete tumor ablation efficacy [111]. In another group, Perlman et al. fabricated PDA composite embedded with gold nanoparticles and copper species by utilizing the advantages of the PDA surface, which works as a reducing agent for both Au and Cu [75]. This multifunctional PDA composite embedded with Au and Cu has the capabilities of serving as both CT and MRI contrast agents [75]. Au-coated PDA nanoparticles can advance applications in the field of image-guided radiation therapy as Au is known as both a radiosensitizer and an imaging contrast agent due to its unique optical properties, surface plasmon resonance properties, and photo-electric effect properties, which open up a strong possibility to apply Au-coated PDA nanoparticles in image-guided combined immuno and radiation therapy to treat cancer efficiently with minimum side effects [75,115]. Image-guided combined-radiation therapy and immunotherapy using PDA nanoparticles may offer maximal control of local and metastatic cancerous lesions while sparing healthy tissue [116,117]. Taken together, there are plenty of research opportunities regarding the potential of using PDA in immunotherapy and radiation therapy that have not been explored yet.

## 4. Conclusions and Future Direction

In the development of innovative cancer treatments, PDA nanoparticles are gaining increasing attention, as they have been found to play a crucial role in various modes of cancer treatments due to their multifunctional properties. PDA nanoparticles have shown their potential in the delivery of anticancer drugs and molecules in many studies and have been used as image-contrast agents due their excellent surface functionalization properties. Considering the impact of the shape of PDA nanoparticles, their differences in surface morphology, and their relative advantages over the regular spherical ones, PDA bowl-shaped mesoporous nanoparticles can be considered as very promising candidates for combining different treatment modalities to overcome the current limitations of nanoparticle-based cancer treatment, including multidrug resistance. Results from studies on HeLa cells demonstrate significant potential for cervical cancer treatment in addition to promising studies involving lung and colon cancer. Moreover, based on previous studies, they have shown efficiency in killing nearly 100% of cancer cells by combined chemo- and photothermal treatment in HeLa cells. The different studies open the door to many opportunities for using PDA nanoparticles to enhance cancer treatment. However, there are limited numbers of in vivo work published on PDA nanoparticles. Considering the promising in vitro results and a few in vivo investigations on PDA bowl-shaped mesoporous nanoparticles, it can be said that they have potential to be ideal candidates in the development of advanced and innovative nanomedicines to treat cancer; therefore, an extensive number of in vivo studies need to be performed in this field. Future research opportunities include studies on the size-depended retention effects of PDA bowl-shaped mesoporous nanoparticles in animals. Other studies are needed to examine degradability, as well as pharmacokinetics studies of PDA mesoporous nanobowls and other promising structures. Research opportunities for enhancing radiotherapy, immunotherapy or their combination are particularly exciting, namely, developing innovative models of cancer treatment to overcome the current limitations in cancer therapy for both localized and metastatic disease.

## Data Availability

Any data included in the manuscript is adequately referenced and available.
